# Advanced Hybrid Closed Loop users’ satisfaction of telemedicine and telenursing in pediatric and young adult type 1 diabetes

**DOI:** 10.3389/fpubh.2023.1249299

**Published:** 2023-08-29

**Authors:** Marta Bassi, Francesca Dufour, Marina Francesca Strati, Daniele Franzone, Marta Scalas, Barbara Lionetti, Giordano Spacco, Francesca Rizza, Prisca Sburlati, Emilio Casalini, Stefano Parodi, Giuseppe d’Annunzio, Nicola Minuto

**Affiliations:** ^1^Pediatric Clinic, IRCCS Istituto Giannina Gaslini, Genoa, Italy; ^2^Department of Neuroscience, Rehabilitation, Ophthalmology, Genetics, Maternal and Child Health (DINOGMI), University of Genoa, Genoa, Italy; ^3^Epidemiology and Biostatistics Unit, Scientific Directorate, IRCCS Istituto Giannina Gaslini, Genoa, Italy

**Keywords:** type 1 diabetes mellitus, telemedicine, telenursing, Advanced Hybrid Closed Loop, insulin infusion set, continuous glucose monitoring

## Abstract

**Background and aims:**

The aim of the study was to evaluate the satisfaction of the use of telemedicine and telenursing in children and young adults with Type 1 Diabetes (T1D) using Advanced Hybrid Closed Loop systems (AHCL) with a focus on the role of connectivity, data download and the ease of technical steps in the set and sensor change procedures.

**Methods:**

An online anonymous survey was administered to AHCL users. The questionnaire consisted of five Clusters: Cluster A-B-C included questions related to the general satisfaction in the use of telemedicine, Cluster D was focused on the role of data download and connectivity, Cluster E was related to satisfaction in telenursing and Cluster F to the perception of ease of execution of the technical steps like changing the infusion set and the sensor.

**Results:**

We collected 136 completed questionnaires. 83.8% of AHCL users were overall satisfied with the quality of the telemedicine service. 88.2% of patients downloaded AHCL data before visits and the overall quality of televisits (data sharing, connectivity, ease of use) was satisfactory for 85.3% of users. Telenursing support during set and sensor change procedures was considered effective by 98% of AHCL users. The sensor and insulin infusion set change procedure is perceived as different for the two systems: set change simpler for Medtronic (*p* = 0.011) users, while sensor change was simpler for Tandem users (*p* = 0.009).

**Conclusion:**

Telemedicine and telenursing have an essential role in diabetology and are highly appreciated in AHCL users. The nurse support in the education of the use of AHCL systems is effective and must be implemented. Unfortunately, not all patients have the technological tools needed for downloading data at home and using telemedicine services; this represents an important challenge for the future of diabetology and for the equity in accessibility to care.

## 1. Introduction

Telemedicine refers to a set of innovative technologies and processes useful to allow remote communication between healthcare professionals and patients (1, 2). This method of visit was implemented and accelerated during the Covid-19 pandemic, where it was essential to continue regular follow-up of chronic diseases, respecting the standards of distance required at the time. Social isolation highly influenced patient care around the world, favoring remote consultation through telehealth/telemedicine as an option to maintain assistance to patients with chronic disease (3). The pandemic accelerated the development of telenursing as a part of telemedicine that focuses on the delivery of care services in the nursing field (4). Digital transformation is already ongoing in pediatrics (5, 6) and many studies have reported the usefulness and the satisfactions of patients in the various fields of pediatrics (7).

Type 1 Diabetes (T1D) is one of the most suitable chronic diseases for this innovation of care thanks to advanced technology systems and innovative devices such as continuous glucose monitoring (CGM) and advanced hybrid closed-loop systems (AHCLs) that allow online data sharing (8). The sharing of the data remotely makes it possible to monitor the patient’s glycemic control and to make any changes to insulin therapy via telehealth services. During Covid-19 pandemic many pediatric diabetes centers adapted to the pandemic by resorting to telemedicine (9, 10). In the last few years, telenursing services dedicated to patients with T1D have increased, to support both correct glycemic monitoring and correct use of advanced insulin pumps (11, 12).

Telemedicine proved to be effective and not inferior to face-to-face visits in maintaining or improving glycemic control in pediatric patients affected by T1D (13–16). Despite the barriers encountered in implementing this service, telemedicine is essential as an alternative follow-up tool for a chronic disease such as diabetes (17–21). Many healthcare professionals of the diabetes teams have been satisfied with the use of telemedicine in patients with T1D and consider it a clinical practice to be strengthened in the future (22). Above all, the patients and their families were satisfied with the use of telemedicine (22, 23).

In a previous work we evaluated the satisfaction of patients and their families in the use of telemedicine through a questionnaire already validated and adapted to T1D patients (12, 24). It was the first survey focused on pediatric and young population affected by type 1 diabetes. The results of the survey demonstrated that telemedicine and telenursing have a positive impact on the daily life of T1D patients and their parents. Data collected showed excellent satisfaction of the service provided, especially in pump users and in patients who live furthest from the center. Furthermore, telenursing service resulted in an effective and appreciated tool to provide education and practical support in the management of insulin pumps and sensors. However, a limitation of the previous study was the absence of questions related to some fundamental aspects in the use of telemedicine, such as device connectivity, data download, quality of video-call and internet connection. After the Covid-19 pandemic we continued to use telemedicine in the Regional Pediatric Diabetes Center of IRCCS Istituto Giannina Gaslini as an alternative follow-up tool in patients who wanted to and for whom remote data sharing was possible. In recent years, the telemedicine service has been officially recognized by the Hospital and the Region and the platforms have been implemented. To date, our Center performs half of the outpatient visits via telemedicine (about 100–150 visits per month) and provides telenursing education in the first week after starting the insulin pump for the support in set and sensor change procedure.

To implement the previous study and to overcome the limitations we decided to investigate fundamental aspects omitted in the previous study such as connectivity and data download. Furthermore, we considered it essential to further investigate patient AHCL systems users of satisfaction in telenursing.

## 2. Methods

### 2.1. Aims and study design

The primary aim of the study was to evaluate the satisfaction of the use of telemedicine and telenursing in children and young adults with T1D using AHCL systems and followed by the Regional Pediatric Diabetes Center of IRCCS Istituto Giannina Gaslini, Genoa, Liguria, Italy. The secondary aims were to assess satisfaction of nursing support in sensor and infusion set change procedures and to assess patients’ perception of ease of performing these procedures, also in relation to the type of AHCL system used.

AHCL initiation training program conducted by healthcare professionals of our Center consists of a theoretical part on the correct use and functioning of the advanced insulin pump (conducted by the diabetologist and the dietician) and a practical part on the correct preparation and placement of infusion set and sensor (conducted by the nurse). In the days following the placement of AHCL system, the first change of CGM sensor and the first change of the infusion set can be assisted by the nursing staff through the telenursing service. Telenursing support is offered to all patients, but those who perform the first sensor change in telenursing aren’t many, because many patients already have the sensor in use from onset and are already able to perform the replacement independently.

The study was conducted from September to December 2022 and consisted of two different phases: the creation and validation of the questionnaire, and its administration to the patients and their families.

### 2.2. Validation of the questionnaire

A new questionnaire was created starting from the one used in the previous study (12), which has been better adapted to AHCL users and implemented to create a new evaluation tool more focused on connectivity, data download and set and sensor change procedures. Content validation of the new questionnaire was performed by a group of six experts in the field of diabetes working at IRCCS Istituto Giannina Gaslini (a pediatric diabetologist, a resident in pediatrics, a psychologist and three pediatric nurses). The content validity was completed after one round only: after the first round, all items had a 100% item-content validity index (I-CVI) for the relevance. Regarding the comprehensibility, two items reached 83.3% of I-CVI, while the remaining 37 had 100% I-CVI (scale-content validity index, S-CVI = 94.9%) (25).

The validated questionnaire consisted of six sections (Supplementary Table S1):

- *Cluster A – Adequacy of medical care*
- *Cluster B – Psychological impact of telemedicine*
- *Cluster C – Possible advantages and future use of telemedicine*
- *Cluster D (**new**) – Connectivity and data download*
- *Cluster E – Telenursing* (satisfaction with the telenursing service was assessed in patients who performed the first nurse-assisted infusion set change)
- *Cluster F (**new**) – Infusion set and glucose sensor replacement*

In all the clusters, responses were given on a 10-point Likert scale ranging either from “strongly disagree” to “strongly agree” or from “extremely difficult” to “extremely easy,” subsequently divided into three sections: 0 to 6 (neutral or dissatisfied/neutral or difficult), 7 to 8 (satisfied/easy) and 9 to 10 (extremely satisfied/extremely easy). A 10-level index of the overall ease of infusion insulin set change was obtained averaging the related answers to the corresponding items in cluster F (rounded to the nearest whole number). Cluster D and Cluster E included some multiple-choice questions (Yes or No). The answers of Cluster A-B-C were also compared based on the age of the patient, the answers of Cluster D and E were also compared based on the type of AHCL used by the patient.

### 2.3. Study population

Participation in the study was voluntary, and completing the survey implied a participant’s consent. The inclusion criteria were: T1D according to the American Diabetes Association (ADA) criteria, age between 1 and 25 years, use of AHCL system for at least 1 month, use of the telemedicine service at least once. Patients and caregivers who were unable to understand, read or write in Italian were excluded. The two AHCL systems used by our patients at the time of the study were Tandem Control-IQ (Tandem Diabetes Care, San Diego, CA, United States) and Minimed 780G (Medtronic, Northridge, CA, United States) (7). The Italian national health system allows AHCL to be prescribed and reimbursed to all patients with T1D. Therefore, our center proposes the use of these advanced systems regardless of the socio-economic situation of the family.

The study was proposed to patients (and their parents/caregivers) who met inclusion criteria during the scheduled visits. The questionnaire was administered online and anonymously. One individual per family answered the questionnaire based on the age or the child’s level of independence: a parent/caregiver answered for patients <12 years of age, while the patient answered for children and young adults ≥12 years of age.

### 2.4. Data analysis

Content validation of the questionnaire was performed using the Content Validity Index for each item (I-CVI) and for the whole questionnaire (“scale validity index,” S-CVI) and then calculated as the proportion of experts providing a positive judgment about both the relevance and the comprehensibility of each item. An item was considered as validated if an I-CVI > 83% was assigned for both the relevance and the comprehensibility, while the corresponding cut-offs for S-CVI were set at 90% (25).

The validated questionnaire was analyzed using absolute frequencies and percentages to summarize qualitative variables. Ten-level Likert scales were aggregated into three categories (0–6, 7–8, and 9–10). The comparison between groups was performed by the Pearson chi-square test or the Fisher exact test when appropriate. All analyses were carried out using the software STATA for Windows, version 13.1 (Stata Corporation, College Station, Texas, United States).

## 3. Results

The survey was administered to 180 patients. We collected 136 completed questionnaires: 41 (30.1%) were filled out by parents or caregivers since the age of the patients was <12 years and 95 (69.9%) by the patients ≥12 years of age. Eighty patients (58.8%) used Tandem Control-IQ and 56 (41.2%) used Minimed 780G. Data related to the responses of Clusters A, B, and C are shown in Table 1.

**Table 1:** Participants responses to questions of Cluster A (adequacy of medical care), Cluster B (psychological impact of telemedicine), and Cluster C (possible advantages and future use of telemedicine).

	>12 years			<12 years			*p*	Total		
	Score 0–6 *N* (%)	Score 7–8 *N* (%)	Score 9–10 *N* (%)	Score 0–6 *N* (%)	Score 7–8 *N* (%)	Score 9–10 *N* (%)		Score 0–6 *N* (%)	Score 7–8 *N* (%)	Score 9–10 *N* (%)
A1. I was able to explain my medical problems well enough via televisit	13 (13.68)	30 (31.58)	52 (54.74)	2 (4.88)	6 (14.63)	33 (80.49)	**0.017**	15 (11.03)	36 (26.47)	85 (62.5)
A2. The absence of physical contact during televisit was not a relevant problem	30 (31.58)	27 (28.42)	38 (40)	6 (14.63)	15 (36.59)	20 (48.78)	0.120	36 (26.47)	42 (30.88)	58 (42.65)
A3. Overall, I am satisfied with the quality of the service provided via televisit	16 (16.84)	26 (27.37)	53 (55.79)	6 (14.63)	11 (26.83)	24 (58.54)	0.938	22 (16.18)	37 (27.21)	77 (56.62)
B1. I was easily able to talk with the medical team during the televisit	14 (14.74)	37 (38.95)	44 (46.32)	4 (9.76)	10 (24.39)	27 (65.85)	0.127	18 (13.24)	47 (34.56)	71 (52.21)
B2. I felt at ease when communicating with my medical team	13 (13.68)	24 (25.26)	58 (61.05)	5 (12.20)	5 (12.20)	31 (75.61)	0.195	18 (13.24)	29 (21.32)	89 (65.44)
B3. I received adequate attention during televisit	9 (9.47)	29 (30.53)	57 (60.00)	6 (14.63)	4 (9.76)	31 (75.61)	**0.026**	15 (11.03)	33 (24.26)	88 (64.71)
B4. I perceived telemedicine as an attention toward me	16 (16.84)	24 (25.26)	55 (57.89)	5 (12.20)	7 (17.07)	29 (70.73)	0.367	21 (15.44)	31 (22.79)	84 (61.76)
C1. I think that televisits are an adequate modality of assistance for my disease	28 (29.47)	34 (35.79)	33 (34.74)	9 (21.95)	9 (21.95)	23 (56.10)	0.064	37 (27.21)	43 (31.62)	56 (41.18)
C2. I am willing to continue some of my follow-up visits via videocall, keeping appointments in person at longer intervals	26 (27.37)	22 (23.16)	47 (49.47)	6 (14.63)	16 (39.02)	19 (46.34)	0.100	32 (23.53)	38 (27.94)	66 (48.53)
C3. Televisits allow me and my family to save time/money/time off work and/or school	12 (12.63)	18 (18.95)	65 (68.42)	3 (7.32)	12 (29.27)	26 (63.41)	0.376	15 (11.03)	30 (22.06)	91 (66.91)

Bold values = statistically significant.

### 3.1. Cluster A—adequacy of medical care

Most patients felt comfortable or very comfortable (respectively 26.5 and 62.5%) to explain their medical problems during televisits. Patients <12 years seem to be able to express their medical problems better than patients ≥12 years (80.5% vs. 54.7%, *p* = 0.017). The absence of physical contact was not a relevant problem for most of the participants (73.5% of score > 6) even if adolescents and young adults ≥12 years suffered the distance more than parents/caregivers (31.6% vs. 14.6% of score 0–6). In conclusion, 83.8% of the population was overall satisfied with the quality of the service provided. Regardless of age, 56.6% was highly satisfied (score 9–10) and 27.2% satisfied (score 7–8) (Table 1).

### 3.2. Cluster B—psychological impact of telemedicine

Most of the population was able to speak easily to the diabetes medical team during the televisits: 52.2% report that they were able to communicate very well (score 9–10) and 34.6% well (score 7–8). 86.8% of responders felt psychologically comfortable when communicating with the medical team (65.4% of them felt very comfortable, score 9–10). There were no significant differences by age in both items B1 and B2. 64.7% were extremely satisfied with the attentions received during telemedicine follow-up visits (score 9–10), even if parents/children <12 years of age were more satisfied than adolescents (75.6% vs. 60.0%, *p* = 0.026). Finally, a large part of the participants perceived telemedicine as an attention toward themselves (Table 1).

### 3.3. Cluster C—possible advantages and future use of telemedicine

Telehealth is not uniformly considered as an appropriate modality of care in young T1D patients: 27.2% of them do not consider it appropriate, 31.6% consider it a moderately appropriate modality (score 7–8) and 41.2% very appropriate (score 9–10), but statistical significance was borderline (*p* = 0.064). 48.5% of responders strongly agree on continuing to be followed via telemedicine (score 9–10), 27.9% seems to want it even if less strongly (score 7–8), while 23.5% prefer face-to-face visits (score 0–6): this desire emerged particularly in the population of adolescents and young adults ≥12 years old (27.4% vs. 14.6%), although the results are not statistically significant (*p* = 0.100). Most of the participants (89% of score > 6) affirmed that televisits allow them to save money and time, avoiding taking time off work and/or school (Table 1).

### 3.4. Cluster D—connectivity and data download

Data of the responses of Cluster D are shown in Table 2. Approximately 88% of patients download data or verify that they are available to the diabetes team before televisits, with no significant differences between the two AHCL systems used (88.75% of Tandem users and 87.50% of Minimed users). Nearly 38.8% of patients using Tandem Control-IQ found downloading data difficult before televisits compared to 16.1% of patients using Minimed 780G (*p* = 0.003). Most of the participants found it easy to share the data and discuss it with the medical team during televisits. Connectivity during televisits was very satisfactory for 41.2% of the population, satisfactory for 38.2% and unsatisfactory for 20.6%. The global quality of the service (data sharing, connection, ease of use) was positively perceived and more than 80% of responders were satisfied with this service.

**Table 2:** Participants responses to questions of Cluster D (connectivity and data download).

	Score 0–6 *N* (%)	Score 7–8 *N* (%)	Score 9–10 *N* (%)	*p*	
D2. It was easy to download the daùta or check data availability before the televisit	31 (38.75)	22 (27.50)	27 (33.75)	**0.003**	Tandem
	9 (16.07)	13 (23.21)	34 (60.71)		Medtronic
	40 (29.41)	35 (25.74)	61 (44.85)		Total
D3.It was easy to share the data and discuss it with the diabetes team during the televisit	19 (23.75)	25 (31.25)	36 (45.00)	0.266	Tandem
	9 (16.07)	14 (25.00)	33 (58.93)		Medtronic
	28 (20.59)	39 (28.68)	69 (50.74)		Total
D4. The connectivity during the televisit was satisfactory	16 (20.00)	29 (36.25)	35 (43.75)	0.761	Tandem
	12 (21.43)	23 (41.07)	21 (37.50)		Medtronic
	28 (20.59)	52 (38.24)	56 (41.18)		Total
D5. The overall quality of the televisit (data sharing, connection, ease of use) was satisfactory	15 (18.75)	30 (37.50)	35 (43.75)	0.216	Tandem
	5 (8.93)	27 (48.21)	24 (42.86)		Medtronic
	20 (14.71)	57 (41.91)	59 (43.38)		Total

Bold values = statistically significant.

### 3.5. Cluster E—telenursing

48 (35%) of 136 patients participating on the survey made the first sensor change assisted via telenursing, of whom 25 (31.2%) were Tandem Control-IQ users and 23 (41.1%) were Minimed 780G users. 63 (46.3%) of 136 patients made the first change of the insulin infusion set assisted via telenursing, of whom 40 (50.0%) were Tandem Control-IQ users and 23 (41.1%) were Minimed 780G users. The support of the nurse was globally considered effective by almost all patients (87.3% score of 9–10, 11% score of 7–8). 54 (85.7%) out of 63 patients considered (score 9–10) the skills acquired during the first infusion set change more than enough and only 2 patients reported that they needed other appointments to learn how to change sets on their own.

### 3.6. Cluster F—infusion set and glucose sensor replacement

**Figure 1:**
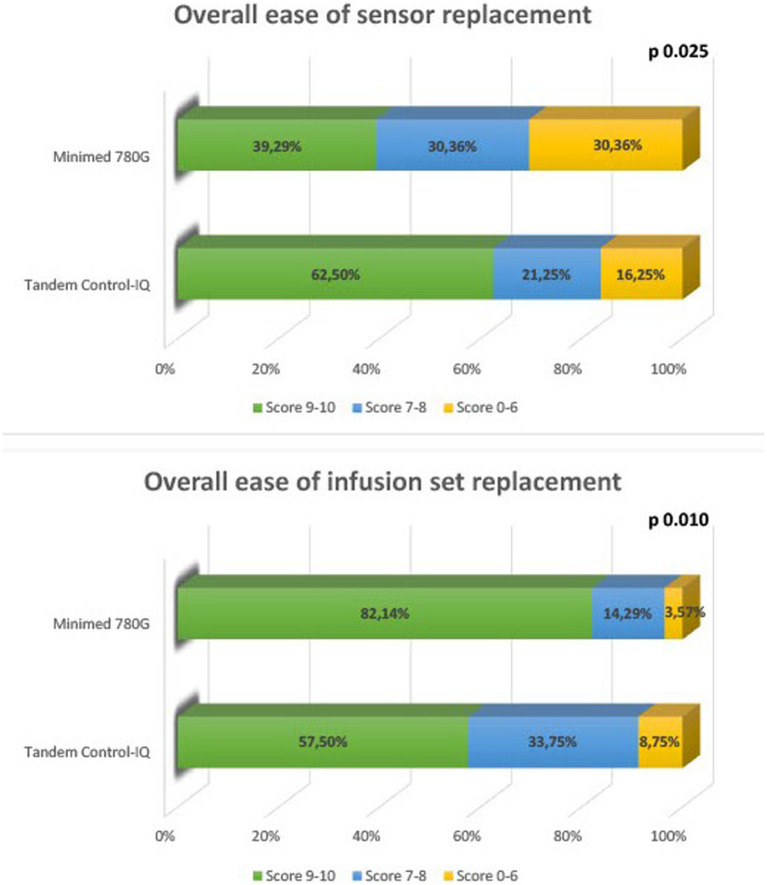
Overall ease of infusion set, and sensor replacement perceived by the patients.

Data regarding the perception of the difficulty in performing the single steps of the infusion set change and the sensor change are shown in Table 3. 47.8% of the patients found filling the tank very easy, however the procedure was simpler in Minimed 780G users than in Tandem Control-IQ users (66.1% vs. 35.0% score 9–10, *p* < 0.001). Connecting the reservoir to the catheter, catheter filling and following the steps indicated by the pump were very easy for most of the patients (respectively 73.5, 77.2, and 73.5%), regardless the insulin pump used. Data showed that the insertion of the cannula subcutaneously was much easier for those who used Minimed 780G rather than Tandem Control-IQ (71.4% vs. 35.0% of score 9–10, *p* < 0.001). Combining the scores of the single steps the infusion set change is globally considered very easy by 67.7% of the patients, with a significant difference between Tandem Control-IQ and Minimed 780G users (57.7% vs. 82%, *p* = 0.011). On the other hand, the glucose sensor replacement is globally considered very easy by 52.9% of patients, with a significant difference between Tandem Control-IQ and Minimed 780G users (62.5% vs. 32.3%, *p* = 0.025). Figure 1 shows the overall ease of infusion set and sensor replacement perceived by the patients.

**Table 3:** Patients’ perception of the difficulty of performing the glucose sensor and the infusion set change steps.

	Score (0–6) *N* (%)	Score (7–8) *N* (%)	Score (9–10) *N* (%)	*p*	
F1. How do you rate the ease of replacing the glucose sensor?	13 (16.25)	17 (21.25)	50 (62.50)	**0.025**	Tandem
	17 (30.36)	17 (30.36)	22 (39.29)		Medtronic
	30 (22.06)	34 (25.00)	72 (52.94)		Total 136
F2. How easy did you find filling the tank?	24 (30.00)	28 (35.00)	28 (35.00)	**<0.001**	Tandem
	2 (3.57)	17 (30.36)	37 (66.07)		Medtronic
	26 (19.12)	45 (33.09)	65 (47.79)		Total 136
F3. How easy did you find it to connect the reservoir to the catheter?	6 (7.50)	19 (23.75)	55 (68.75)	0.332	Tandem
	2 (3.57)	9 (16.07)	45 (80.36)		Medtronic
	8 (5.88)	28 (20.59)	100 (73.53)		Total 136
F4. How easy did you find catheter filling?	6 (7.50)	16 (20.00)	58 (72.50)	0.338	Tandem
	2 (3.57)	7 (12.50)	47 (83.93)		Medtronic
	8 (5.88)	23 (16.91)	105 (77.21)		Total 136
F5. How easy did you find the placement of the cannula subcutaneously?	18 (22.50)	34 (42.50)	28 (35.00)	**<0.001**	Tandem
	1 (1.79)	15 (26.79)	40 (71.43)		Medtronic
	19 (13.97)	49 (36.03)	68 (50.00)		Total 136
F6. How easy did you find it to follow the steps given by the pump?	6 (7.50)	20 (25.00)	54 (67.50)	0.171	Tandem
	2 (3.57)	8 (14.29)	46 (82.14)		Medtronic
	8 (5.88)	28 (20.59)	100 (73.53)		Total 136
FF. Ease of replacing the infusion set (calculated)	7 (8.75)	27 (33.75)	46 (57.50)	**0.010**	Tandem
	2 (3.57)	8 (14.29)	46 (82.14)		Medtronic
	9 (6.62)	35 (25.74)	92 (67.65)		Total 136

Bold values = statistically significant.

## 4. Discussion

The use of telemedicine in the care of T1D pediatric patients has undergone a strong implementation since the Covid-19 pandemic.

A cross-sectional electronic survey distributed through a global network during the pandemic showed that the proportion of people with diabetes receiving telemedicine visits increased from <10 to >50% (21). Even before the pandemic, Wood et al. had shown that telehealth improved adherence to ADA recommendations increasing the number of follow-up visits (2.0 ± 1.3 times per year in the year prior to starting telemedicine and 2.9 ± 1.3 times, in the year after starting telemedicine, *p* < 0.0001), proving to be equivalent to in-person visits to maintain glycated hemoglobin (HbA1c) levels (13). To date, telemedicine continues to be used effectively on glycemic control and satisfactory for the patient in many countries (12, 22, 23). Several studies have demonstrated an improvement in CGM parameters in patients followed with telemedicine service during or after the pandemic (15, 16). In a recent study 28 children with T1D and their caregivers have carried out remote visits for 6 months. After 3 and 6 months of remote visits, Time in Range and Time Above Range significantly improved just as their psychological health (19). However, in low-middle income countries and in rural areas telemedicine services where used, have proved useful in maintaining regular patient follow-up but not always effective in maintaining a good glycemic control (17, 18). The role of telehealth, in these areas where technology is less used, may be fundamental to decrease clinical costs through the prompt diagnosis of decompensation, fewer visits to the emergency room for complications like ketoacidosis and severe hypoglycemia, and healthier lifestyle behaviors (26).

The barriers in the use of telemedicine in T1D care have been extensively analyzed and the aspect of connectivity and access to technology represents one of the essential points for the correct use of this service (19–21). Starting from this consideration, we wanted to evaluate the satisfaction in the use of telemedicine, with a particular focus on the aspect of connectivity, missing in the previous study but fundamental in evaluating the patient experience. Patients using AHCL systems were the most satisfied of the telemedicine service in our previous study (12). In a country like Italy, patients treated with highly technological instruments can benefit most from remote visits thanks to the possibility of comprehensive glycemic and insulin pump data sharing via dedicated cloud platforms.

A patient followed up at our center has an average of two televisits and two in-person visits per year. We also decided to include in the study the patients who have used it less (at least once), in order to avoid the bias of excluding those who have discontinued using the service even after only one visit due to dissatisfaction with the telemedicine. Despite the general satisfaction with the telemedicine service in AHCL users, parents or caregivers seem to be more satisfied than the patients in some aspects of the adequacy of care and psychological impact. Data showed that both young patients ≥12 years and parents/caregivers of patients <12 years were able to express their medical problems during televisits, but it seems easier for parents than for young T1D patients. Most of the participants declared that they receive adequate attention from the healthcare professionals, but the parents perceive more attention than the children and young patients. Patients and parents speak easily with the medical team, felt comfortable during televisits, perceived remote visits as an attention toward them and an adequate modality of assistance for T1D. The absence of physical contact was not a relevant problem for most of the participants even if adolescents and young adults suffered the distance more than parents (14.6% vs. 31.6% of disagreement scores), although this data was not statistically significant. Most of the participants will continue to use telemedicine, but the preference for in-person visits emerged particularly in the patients (27.4% vs. 14.6% of disagreement scores). Saving time and money are confirmed factors of satisfaction, even if this aspect seems less important for the patient (12.6% vs. 7.3% of disagreement score). Globally and regardless of age, 83.8% of the population was overall satisfied with the quality of the service provided.

These discrepancies between the perception of children and parents or caregivers is not surprisingly both because they are in line with the results of the previous study and because of the well-known importance of the relationship between healthcare professional and patient in chronic diseases, especially in the pediatric age (12, 27, 28). We therefore believe it is normal that an adolescent or young adult, even if largely satisfied with the telemedicine service, suffers more from the lack of physical contact, has more difficulty explaining their problems and perceives to receive less attention from the healthcare professional remotely. In the light of these results and considering outdated the limitations relating to the pandemic period, we believe it is essential to evaluate the benefits and critical issues in the use of telemedicine patient by patient. In fact, only by evaluating all the characteristics and needs of the patient (i.e., age, psychological and therapeutic situation, distance from the clinic, economic conditions of the family, ability to use data sharing platforms) it is possible for the diabetes team to choose the best modality of assistance for each patient and in every moment of his therapeutic path. Data relating to the distance from the diabetes center had already been collected in the previous study which demonstrated the greater satisfaction of those who lived further away from the clinic. Given that AHCLs are used by our patients regardless of socio-economic status, we suppose that the results relating to the study population of the previous study are representative of that of AHCL users.

Data download and sharing are fundamental aspects of the success of the televisit at our Center, which consists of a face-to-face remote visit on the company’s online videocall platform during which glycemic and insulin pump data are discussed, sharing in real-time the data download screen. Despite most patients (88.8%) declare that they download data before the visit, 11.2% of them do not, declaring to encounter various kinds of technical or connection problems. This percentage is not negligible, because it means that 1 out of 10 patients is unable to carry out a complete and effective televisit according to our standards. It would be important to understand whether the failure to download and share data is due to forgetfulness or negligence of the patient or to the lack of suitable technological tools to carry it out. Comparing the two AHCL systems, Minimed 780G users download data more easily than Tandem Control-IQ users. This is an obvious result since the download on the Minimed 780G platform (Carelink^®^) is based on an automatic update while the download on the Tandem Control-IQ platform (Glooko^®^) requires the connection of the insulin pump to a suitable electronic device. A further aspect to underline is 20.6% of patients are dissatisfied with the quality of the connection during the televisits. This is a significant percentage that highlights how much work still needs to be done in improving the telehealth platforms and the connections made available by the Institutions.

Thanks to the support of the regional Association for families of T1D patients (ADG Genova Onlus), our center is implementing the use of technology and telemedicine providing free technological devices and connection to families who are not economically able to buy them independently. However, we believe that much more can still be done in both our center and in other centers of high-income countries, also with the support of companies producing systems that require advanced technological tools available for the best T1D care without discrimination.

Telenursing was confirmed to be effective for patients and parents/caregivers also in this second survey dedicated to AHCL users. We chose to evaluate telenursing satisfaction only in patients who made their first infusion set change remotely, because it is a procedure that requires many steps and it allows a better evaluation of the efficacy of the nurse’s support. The support of the nurse is considered effective by 98.4% of patients and 96.8% of patients did not need other appointments to learn how to do the insulin set change. Given the excellent results relating to its use, our diabetes team is strongly motivated to implement and improve the telenursing service.

In the survey, we decided to evaluate in detail the difficulty of the single steps of the insulin infusion set change to identify the problematic issues and implement the nursing support in the most critical steps for each AHCL system. According to the results of the survey, the filling of the tank and the placement of the cannula emerge as the most critical steps. The greatest difficulties in these two steps were encountered by the Tandem Control-IQ Users (difficulty of filling the tank 28.7% vs. 3.3%, *p* < 0.001; difficulty of placement of the cannula 20.7% vs. 1.6%, *p* < 0.001). These results are consistent with the technical characteristics of the two instruments. In fact, filling the tank of the Tandem Control-IQ requires the air to be aspired from the tank before refilling as an additional step. Even the placement of the subcutaneous set of Tandem Control-IQ (Autosoft 90 or 30) requires some additional steps compared to Minimed 780G (unwinding the catheter and manual loading of the needle). In the case of the glucose sensor, as expected, the multi-step replacement of the Guardian sensor of Minimed 780G is perceived as more complicated than the single-step procedure of the Dexcom sensor of Tandem Control-IQ. Since it is obvious that multi-step procedures can be more complicated to perform by the patient, it is essential that the nurse gives more support to the patients during these most critical steps.

A limitation of this study is that we included only AHCL users in the survey, thus selecting the study population and encouraging the participation of patients and families who are more inclined and capable with technology. Furthermore, the restriction of the survey to a cohort of T1D patients followed by a single center of a high-income country limits the reproducibility of the results. Another limitation is related to the anonymous online administration which was not a guarantee of completion by all patients/parents who had consented to participate and did not allow the collection of clinical data of the study population. Furthermore, the number of televisits performed by the participants, the number of patients discontinuing early the service were not available for evaluation. Although AHCL are used by our patients regardless of socioeconomic status, the lack of these data can also be considered a limitation of this study. The strength of our study is that, to our knowledge, it is the first survey on satisfaction of telemedicine with a dedicated focus on connectivity and data download, which are well recognized as barriers and key factors in the use of telemedicine (21). Moreover, this is the first survey that evaluates in detail the difficulties encountered by patients in using AHCL in terms of set and sensor replacement, allowing diabetes teams to identify the critical steps to better direct the support to the patient.

## 5. Conclusions and future perspectives

This study showed once again the satisfaction of T1D patients and their parents assisted with telemedicine service. The survey also assessed the download and sharing of data and the connectivity as critical elements for the effective use of televisits. To perform a successful televisit, the patient must download and share the glycemic and pump data from his/her own device. The results of the study show that a minority of patients do not download data and are not satisfied with the quality of connectivity during the visit. These data underline the need for continued efforts by diabetes centers, patients’ associations, manufacturers of technological device for T1D therapy, hospitals or institutions and healthcare systems to ensure equitable access to technologies and treatments for T1D patients. Data show great satisfaction in telenursing and suggest the importance of implementing this service, dedicating nursing support where the sensor and infusion set replacement multi-step procedures are more difficult for patients. Finally, we hope that this work will be an inspiration for companies that produce AHCL to improve the steps that are considered more critical by patients. The connection of the insulin pump data with phones seems to have become mandatory in order to be able to manage the data with the help of the referring doctors. The simplicity of the steps in positioning the infusion sets and sensors is highly appreciated by patients and could be further simplified in order to reduce errors that could lead to clinical consequences.

## Data availability statement

The original contributions presented in the study are included in the article/Supplementary material, further inquiries can be directed to the corresponding author.

## Author contributions

MB and FD designed the study and wrote the manuscript. MFS reviewed the manuscript and contributed to the discussion. DF wrote the manuscript and designed the tables. MS wrote the manuscript and designed the figures. BL and GS researched data and wrote the manuscript. FR designed the study. PS researched data. EC contributed to the creation and validation of the questionnaire. SP performed statistical analyses. Gd’A reviewed the manuscript. NM designed the study and contributed to the discussion.

## Conflict of interest

The authors declare that the research was conducted in the absence of any commercial or financial relationships that could be construed as a potential conflict of interest.

## Publisher’s note

All claims expressed in this article are solely those of the authors and do not necessarily represent those of their affiliated organizations, or those of the publisher, the editors and the reviewers. Any product that may be evaluated in this article, or claim that may be made by its manufacturer, is not guaranteed or endorsed by the publisher.
