# Supplementary material for: Advanced Hybrid Closed Loop users’ satisfaction of telemedicine and telenursing in pediatric and young adult type 1 diabetes
Source: Front Public Health. 2023 Aug 29;11:1249299. doi: 10.3389/fpubh.2023.1249299 (PMC10497768; doi:10.3389/fpubh.2023.1249299)
Supplement: Supplementary file 1 [file Table_1.DOCX]

**Supplementary Table 1.** The questionnaire

|  | **Question** | **Score** |
| --- | --- | --- |
| **Cluster A**  Adequacy of Medical care | A1. I was able to explain my medical problems well enough via televisit | 0 - strongly disagree to 10 - strongly agree |
|  | A2. The absence of physical contact during the televisit was not a relevant problem | 0 - strongly disagree to 10 - strongly agree |
|  | A3. Overall, I am satisfied with the quality of the service provided via televisit | 0 - strongly disagree to 10 - strongly agree |
| **Cluster B**  Psychological impact of telemedicine | B1. I was easily able to talk with the medical team during the televisit | 0 - strongly disagree to 10 - strongly agree |
|  | B2. I felt at ease when communicating with my medical team | 0 - strongly disagree to 10 - strongly agree |
|  | B3. I received adequate attention | 0 - strongly disagree to 10 - strongly agree |
|  | B4. I perceived telemedicine as an attention towards me in this period | 0 - strongly disagree to 10 - strongly agree |
| **Cluster C**  Possible advantages and future use of telemedicine | C1. I think that televisits are an adequate modality of assistance for my disease | 0 - strongly disagree to 10 - strongly agree |
|  | C2. I am willing to continue some of my follow-up visits via videocall, keeping appointments in person at longer intervals | 0 - strongly disagree to 10 - strongly agree |
|  | C3. Televisits allow me to save time/money and/or time off work and/or school | 0 - strongly disagree to 10 - strongly agree |
| **Cluster D**  Connectivity and data download Telenursing | D1. Do you download the data or check that data are available on the download platforms before the televisit? | Yes or No |
|  | D2. It was easy to download the data or check data availability before the televisit | 0 - strongly disagree to 10 - strongly agree |
|  | D3. It was easy to share the data and discuss it with the diabetes team during the televisit | 0 - strongly disagree to 10 - strongly agree |
|  | D4. The connectivity during the televisit was satisfactory | 0 - strongly disagree to 10 - strongly agree |
|  | D5. The overall quality of the televisit (data sharing, connection, ease of use) was satisfactory | 0 - strongly disagree to 10 - strongly agree |
| **Cluster E**  Telenursing | E1. Did you replace your first glucose sensor in telenursing? | Yes or No |
|  | E2. Did you replace your first insulin infusion set in telenursing? | Yes or No |
|  | E3. The nurse support was effective during televisit | 0 - strongly disagree to 10 - strongly agree |
|  | E4. The nurse support was sufficient to acquire the skills to do the infusion set or sensor change on my own and I wouldn’t need more telenursing appointments | 0 - strongly disagree to 10 - strongly agree |
| **Cluster F**  Infusion set and glucose sensor replacement | F1. How do you rate the ease of replacing the glucose sensor? | 0 – extremely difficult to 10 – extremely easy |
|  | **How would you rate the ease of performing the following steps:** |  |
|  | F2. Filling the insulin reservoir | 0 – extremely difficult to 10 – extremely easy |
|  | F3. Connection of the reservoir to the catheter | 0 – extremely difficult to 10 – extremely easy |
|  | F4. Filling the catheter | 0 – extremely difficult to 10 – extremely easy |
|  | F5. Placement of the cannula in the subcutaneous tissue | 0 – extremely difficult to 10 – extremely easy |
|  | F6. Follow the steps guided by the insulin pump | 0 – extremely difficult to 10 – extremely easy |
|  | **Not in the questionnaire**  FF. Ease of replacing the infusion set | Statistically calculated (see methods) |
